# Peritoneal MSCs-derived exosomes suppress CCL24 synthesis through miR-320d delivery contributing to the improvement of peritoneal dialysis-associated fibrosis

**DOI:** 10.1038/s41598-026-42489-w

**Published:** 2026-03-04

**Authors:** Xinhui Zhao, Minhui Xi, Hualin Qi, Jia Xu, Rui Yuan, Danye Shi, Jingyuan Lu

**Affiliations:** 1 Department of Nephrology, Shanghai Pudong New Area People’s Hospital, Shanghai, 200120 China; 2 Department of Nephrology, Minhang Hospital, Fudan University, Shanghai, China

**Keywords:** Peritoneal dialysis, Fibrosis, CCL24, Mesenchymal stem cells, Exosomes, Cell biology, Diseases, Stem cells

## Abstract

Mesothelial-to-mesenchymal transition (MMT) is a key pathological process driving peritoneal fibrosis in patients undergoing peritoneal dialysis (PD). Although Chemokine ligand 24 (CCL24) is implicated in multi-organ fibrosis, its role in MMT and PD-related fibrosis is still unknown. And the therapeutic potential of peritoneal mesenchymal stem cells (pMSCs) also remains unexplored. To elucidate the mechanistic role of CCL24 in MMT and PD-related fibrosis, and the reversal effects of human pMSCs-derived exosomes (HpMSCs-Exo) loaded with miRNAs, a series of experiments, including qRT-PCR, western blotting, ELISA, immunofluorescence, hematoxylin-eosin, and Masson’s trichrome staining, were employed. In a PD-induced peritoneal fibrosis rat model, CCL24 was significantly upregulated in peritoneal tissues, predominantly localized to macrophages. Macrophage-derived CCL24 promoted MMT via the CCR3/P38 MAPK pathway, an effect reversed by HpMSCs and their exosomes. Mechanistically, HpMSCs-Exo delivered miR-320d into macrophages. KLF7, the target gene of miR-320d, abolished the suppressive effects of miR-320d mimics and HpMSCs-Exo on STAT3 phosphorylation level and CCL24 expression in macrophages. Furthermore, HpMSCs-Exo attenuated MMT and PD-related fibrosis, with miR-320d-enriched exosomes exhibiting superior efficacy. HpMSCs-Exo deliver miR-320d into macrophages, suppressing CCL24 synthesis and secretion via the KLF7/STAT3 pathway, and thereby ameliorating MMT-driven peritoneal fibrosis. Autologous pMSCs-Exo engineered with miR-320d represent a promising therapeutic strategy for halting PD-related fibrosis progression.

**Supplementary Information:**

The online version contains supplementary material available at 10.1038/s41598-026-42489-w.

## Introduction

Peritoneal dialysis (PD) operates on the principles of diffusion and osmosis across the peritoneal membrane, facilitating the exchange of solutes and uremic toxins between systemic circulation and dialysis fluid. As a life-sustaining renal replacement therapy, PD preserves the patient’s residual renal function and currently serves approximately 3.8 million patients with end-stage renal disease globally^1^. Unfortunately, long-term PD inevitably induces structural and functional alterations in the peritoneal membrane, characterized by submesothelial thickening, angiogenesis, and progressive peritoneal fibrosis^2^, which collectively compromise ultrafiltration capacity and solute clearance, ultimately leading to technique failure^3^. Central to peritoneal fibrosis is mesothelial-to-mesenchymal transition (MMT), a pathological reprogramming process wherein peritoneal mesothelial cells (PMC) undergo phenotypic transformation^4^. During MMT, PMCs shed epithelial markers (E-cadherin) and acquire mesenchymal features (α-SMA), concomitant with enhanced extracellular matrix (ECM) deposition^5^. Up to now, there is still a lack of effective treatment regimens for PD-related fibrosis. Consequently, novel therapies targeting MMT are urgently needed to prevent and alleviate the progression of PD-related peritoneal fibrosis.

During the development of MMT, sterile inflammation, especially macrophage function imbalance, has been devoted considerable attention^6^. For instance, interferon-stimulated factors from peritoneal macrophages promote MMT^7^. Moreover, in our previous single-cell transcriptomic analysis, a macrophage subpopulation (Macro4) enriched in chemokine ligand 24 (CCL24) is upregulated in PD-induced peritoneal fibrosis rat model^8^. CCL24, primarily secreted by macrophages^9^, drives fibrosis in multiple organs, such as skin^10^. However, the role of macrophage-derived CCL24 in MMT and PD-associated fibrosis tissues remains unconfirmed.

Mesenchymal stem cells (MSCs), from adipose tissue, bone marrow, or umbilical cord, modulate macrophage function and ameliorate dialysis-induced peritoneal fibrosis^11,12^. Notably, peritoneal MSCs (pMSCs) isolated from PD effluent, exhibit superior reparative capabilities compared to umbilical cord-derived MSCs^13^. Meanwhile, human pMSCs (HpMSCs) reverse the upregulation of Macro4 with high expression of CCL24 in PD-induced peritoneal fibrosis rats^8^, suggesting their capacity to inhibit CCL24 synthesis in macrophages, a mechanism requiring validation. Furthermore, exosomes (MSCs-Exo) are key mediators of MSC paracrine effects, delivering miRNAs to recipient cells. For instance, MSCs-derived exosomal miR-146a-5p inhibits microglia pyroptosis, and engineered exosomes loaded with miR-146a-5p mimic enhance efficacy^14^. Consequently, we speculate that HpMSCs-Exo may deliver miRNAs to macrophages, leading to the improvement of CCL24 synthesis and MMT, and engineered exosomes could offer superior inhibitory effects on PD-related fibrosis.

In the present study, our research objective was to demonstrate the effects of macrophage-derived CCL24 on MMT of PMCs and the inhibitory effects of HpMSCs-Exo. Our findings indicate that HpMSCs-Exo attenuate the synthesis and secretion of CCL24 in macrophages via the miR-320d/KLF7/STAT3 pathway, and then improve MMT and PD-related fibrosis progression. Furthermore, engineered HpMSCs-Exo loaded with miR-320d have stronger inhibitory effects.

## Methods

### Cell culture and treatment

The THP-1 cell line was purchased from Wuhan Procell Life Technology Co., Ltd. (CL-0233). Cells were cultured in RPMI-1640 medium (Gibco, 11875093, Shanghai, CN) supplemented with 10% fetal bovine serum (FBS; Gibco, A5670701), 0.05 mM β-mercaptoethanol (Gibco, 31350010), and 1% penicillin-streptomycin (P/S; Gibco, 15140148) at 37 °C in a humidified incubator with 5% CO₂. To induce polarization into M0-type macrophages, THP-1 cells in the logarithmic growth phase with optimal viability were seeded into 6-well plates at a density of 1 × 10^6^ cells per well, and then treated with 100 ng/ml phorbol 12-myristate 13-acetate (PMA; ABclonal, RPM0015, Wuhan, CN) for 48 h. Subsequently, the PMA-containing medium was replaced with serum-free medium, and cells were rested for an additional 24 h. To obtain M2 macrophages, THP1-derived M0 macrophages were then treated with medium containing IL-4 (ABclonal, RP00995, 20 ng/ml), IL-13 (ABclonal, RP01320, 20 ng/ml)^15^, and exosome-free FBS for 48 h.

Primary rat peritoneal macrophages were isolated from unstimulated rats via peritoneal lavage^16^. The entire peritoneal lavage fluid was centrifuged for 5 min. Subsequently, the resulting cell pellet was resuspended in complete RPMI-1640 medium supplemented with 10% FBS, and then incubated at 37 °C in a humidified incubator with 5% CO_2_. Immunofluorescence using a CD68 antibody (Abcam, ab283654, Hangzhou, CN) was conducted to assess the purity of the macrophage populations and showed that 98% of the cells were CD68-positive.

Later, THP1-derived M2 macrophages or primary rat peritoneal macrophages were stimulated with HpMSCs-conditioned medium (HpMSCs-CM) or exosomes-free HpMSCs-CM (HpMSCs-CM^Exo−free^) or HpMSCs-Exo with different concentrations (12.5, 25, 50 µg/ml)^17^. Exosomes-free FBS and HpMSCs-CM^Exo−free^ were achieved by high-speed centrifugation to remove exosomes in serum and HpMSCs-CM, respectively. Meanwhile, THP1-derived M2 macrophages were transfected with siRNA or miRNA in the presence of vectors or KLF7 overexpression plasmids. And the culture supernatant from macrophages with different treatments was collected after removal of cell debris by centrifugation.

HpMSCs were obtained from PD effluents of anonymous patients in our previous study^8^ and frozen in liquid nitrogen with 10% dimethyl sulfoxide (DMSO). The frozen cells were rapidly thawed and washed with culture medium. After washing, HpMSCs were cultured in Dulbecco’s modified Eagle’s medium/Ham’s nutrient mixture F12 (DMEM/F12; Procell, PM150312) containing 10% exosome-free FBS and 1% penicillin/streptomycin at 37 °C in a 5% CO_2_ atmosphere incubator, and expanded by replacing the medium every 3 days until reaching 70%-80% confluence. Then, the culture supernatant was collected as HpMSCs-CM.

Human PMCs (HPMCs) and rat peritoneal mesothelial cells (RPMCs) were purchased from Wuhan Procell Life Technology Co., Ltd. (Catalog numbers: CP-H180 and CP-R157) and cultured in the manufacturer’s specialized medium (Procell, CM-H180 and CM-R157, respectively) at 37 °C with 5% CO₂. HPMCs were treated with the supernatant from M2 macrophages with different treatments or together with pre-treatment of recombinant CCL24 (100 ng/mL^18^, Gibco, 300-33-5UG) or CCR3 inhibitor ALK4290 (5µM^19^, MCE, HY136788, Shanghai, CN) or p38 inhibitor SB203580 (10µM^20^, MCE, HY10256) for 1 h.

### Cell transfection

CCL24 siRNA (SIGS0007801-1), miR-320d inhibitor (miR20006764-1-5), and miR-320d mimic (miR10006764-1-5) were obtained from RIBOBIO (Guangzhou, CN). Synthetic DNA fragments of KLF7 (Vectorbuilder, VB900007-8978mzp, Guangzhou, CN) were inserted into pRP[Exp]-EGFP/Puro-CAG vector. The plasmids were transfected into the cells using Lipofectamine 3000 according to the manufacturer’s protocol (Invitrogen, Carlsbad, USA). After transfection for approximately 72 h, the cells were used for further research.

### Flow cytometry

Cells were stained with the respective fluorochrome conjugated monoclonal antibodies to the cell surface markers as follows: anti-CD206 (Elabscience, E-AB-F1161E, Wuhan, CN), and anti-CD163 (BioLegend, 333617, CA, USA). As a control, cells were stained with mouse IgG1 isotype-control antibodies. Cells were analysed using a FACScan (BD) with CellQuest Analysis (BD Biosciences) and FlowJo software v.8.8 (TreeStar).

### ELISA

The cell supernatants and tissues were collected and analyzed by ELISA using a rat CCL24 ELISA kit (FineTest, ER1168, Wuhan, China), a human CCL24 ELISA kit (Abcam, ab100509), and a human IL-10 ELISA kit (Elabscience, E-EL-H6154).

### qRT-PCR

Total RNA was isolated using TRIzol reagent (Invitrogen, 12183555, CA, USA) according to the manufacturer’s instructions. First-strand cDNA was prepared using a reverse transcription kit (Thermofisher, 4368814, MA, USA) according to the manufacturer’s protocol. SYBR was used for RT-qPCR analysis on the LightCycler 480 (Roche, Basel, CH). After reactions were completed, the relative gene expression levels were calculated using the 2^–ΔΔCt^ method, and GAPDH was used as the endogenous control. The primers used in this study were human CCL24, forward: CCTGTTACCTCCGGGTCCTT, reverse: GAGCCCGTAGGGATGATGTG; human GAPDH, forward: GAAGGTGAAGGTCGGAGTC, reverse: GAAGATGGTGATGGGATTTC; rat CCL24, forward: CAGGCTCCACCACCATCATT, reverse: AAGGTCACGCAGCAAGATGA; rat GAPDH, forward: ACGGGAAACCCATCACCATC, reverse: CTCGTGGTTCACACCCATCA. The PCR reaction involved an initial pre-denaturation step at 95 °C for 2 min, followed by 40 cycles of 95℃ for 15 s, 60℃ for 20 s, and 72℃ for 20 s.

For miRNA detection: cells used for total RNA extraction using miRNeasy Mini Kit (QIAGEN, 217004, CA, USA); 1 µg total RNA was used for reverse transcription. Analysis of miRNAs was done using miScript PCR system (QIAGEN, 218161, CA, USA) by QuantStudio Real-time PCR system version 1.3 (Applied Biosystems, Foster City, CA), and U6 was used for inner control. The primers used in this study were human miR-320d and rat miR-320: AAAGCTGGGTTGAGAG; human miR-148a: TGCCGCTCAGTGCACTACAGAA; human miR-21: TGCGGCTAGCTTATCAGACT; human miR-223: TGCCGCTGTCAGTTTGTCAAAT; human miR-125a: TGCCGCACAGGTGAGGTTCTTG; human miR-125b: GCAGTCCCTGAGACCCT; human miR-1246: TGCGGCAATGGATTTTTGG; universal primer: GTGCAGGGTCCGAGGT; human U6 and rat U6: Forward: CGCTTCGGCAGCACATATAC, Reverse: AAATATGGAACGCTTCACGA. The initial activation was performed at 95 °C for 15 min and followed by 40 cycles, denaturation at 95 °C for 15 s, annealing at 55 °C for 30s, and extension at 70 °C for 30s.

### Western blotting

Proteins were extracted by using RIPA buffer containing a protease inhibitor cocktail (Beyotime, P0013B, Shanghai, CN), measured using a BCA protein detection kit (Beyotime, P0009), separated by 10% SDS-PAGE and transferred onto PVDF membranes (Millipore, IPVH00010, MA, USA). The membrane was blocked with 5% non-fat milk with TBST for 1 h at room temperature and incubated with the appropriate primary antibodies (CCL24, Proteintech, 22306-1-AP, 1:1000, Wuhan, CN; GAPDH, Proteintech, 10494-1-AP, 1:2000; E-cadherin, Proteintech, 20874-1-AP, 1:1000; fibronectin, FN, Proteintech,15613-1-AP, 1:1000; α-SMA, Proteintech, 14395-1-AP, 1:1000; Collagen I, Proteintech, 86093-1-RR, 1:1000; p-p38, Proteintech, 28796-1-AP, 1:1000; p38, Proteintech, 14064-1-AP, 1:1000; p-Akt, Proteintech, 80455-1-RR, 1:1000; Akt, Proteintech, 10176-2-AP, 1:1000; p-ERK, Proteintech, 28733-1-AP, 1:1000; ERK, Abcam, ab184699, 1:1000; CD9, Abcam, ab236630, 1:1000; CD63, Abcam, ab134045, 1:1000; CD81, Abcam, ab219209, 1:1000; Calnexin, Abcam, ab22595, 1:1000; p-STAT3, Abcam, ab267373, 1:1000; STAT3, Abcam, ab68153, 1:1000; KLF7, Abcam, ab197690, 1:1000) overnight at 4 °C. After washing with TBST for three times, the membrane was incubated with HRP-labeled secondary antibodies (Beyotime, A0208) for 2 h at room temperature. The proteins were then examined using ECL reagent (BIO-RAD, 170–5061, Hercules, USA).

### Isolation and measurement of exosome

Exosome was isolated from cell cultured media via three sequential centrifugation steps at 4 °C: (1) 30 min at 2000 × g to remove cells, (2) 45 min at 10,000 × g to remove cell debris and (3) ultracentrifugation at 100,000 × g for 70 min to pellet exosome. The pellet was finally re-suspended in PBS and centrifuged at 100,000 × g for another 70 min to remove soluble and secreted proteins. For exosome observation, exosomes were applied to a copper grid, followed by uranyl acetate (Sigma, 79690, Shanghai, CN), and images were captured using the JEM1230 TEM system (JEOL, Tokyo, Japan).

### Chromatin immunoprecipitations (ChIP)-qPCR assay

THP-1-derived M2 macrophages were plated in 150-cm plates and grown overnight to about 70% confluence. Cells were treated with HpMSCs-Exo for 48 h. Chromatin immunoprecipitations were performed with the SimpleChIP Enzymatic Chromatin IP kit (CST, 9003, Shanghai, CN) following the manufacturer’s instruction. Immunoprecipitation reactions were performed by using antibodies against STAT3 (CST, 12640), and rabbit IgG (CST, 2729). Immunoprecipitated DNA samples were then analyzed by qPCR. The primers for the segment of DNA analyzed for CCL24 binding were as follows: forward primer, 5-GGACTCTTATTGGCCGCCTTCC-3; reverse primer, 5-CGGGCATGGTGACTGGGATTTC-3.

#### Exosome uptake assay

To obtain HpMSCs-Exo loaded with FITC-labeled miR-320d, a total transfection reaction mixture of 150 µl was prepared as follows: 10 µl of Exo-Fect Reagent (SBI, EXFT200A-1), 20 µl of miR-320d (20 pmol) labeled with or without FITC, 70 µl of 1x PBS, and 50 µl of exosomes resuspended in 1x PBS. The mixture was gently mixed and then incubated at 37 °C for 10 min. Subsequently, 30 µl of ExoQuick-TC Tissue Culture Media Exosome Precipitation Solution was added, mixed thoroughly, and incubated for 30 min. The sample was then centrifuged at 12,000 rpm for 3 min. The supernatant was discarded, and the exosome pellet was retained. The prepared HpMSCs-Exo and HpMSCs-Exo-FITC-miR320d were incubated with THP-1-derived macrophages for 24 h. The intracellular distribution of FITC in each group was observed using a confocal laser scanning microscope (Olympus, FV3000, Tokyo, Japan).

#### Luciferase reporter assay

KLF7 3’UTR MUT and KLF7 3’UTR WT were cloned into the luciferase reporter plasmid psiCHECK-2 (Promega). Then, the different luciferase reporter plasmids were transfected into the cells with or without repressed miR-320d mimic using transfection reagent (GenePharma, Shanghai, CN) according to the protocol. 72 h later, the luciferase activities of firefly and Renilla were detected using a luciferase detection kit (Beyotime, RG027).

### Preparation of modified HpMSCs-Exo loaded with miR-320d

HpMSCs were transfected with either miRNA mimics NC or miR-320d mimics using Lipofectamine 3000. After 48 h of transfection, the cells were washed with PBS and replaced with fresh culture medium to remove leftover lipofectamin-miRNA complexes. Then, after 24 h, exosomes were isolated from the cell culture supernatants. The exosomes derived from HpMSCs transfected with miRNA mimics NC served as the control group (control HpMSCs-Exo, abbreviated as Exo-C). The exosomes derived from HpMSCs transfected with miR-320d mimics constituted the experimental group, characterized by a high abundance of miR-320d (modified HpMSCs-Exo, abbreviated as Exo-M).

### Animal experiments

An animal model of PD-induced peritoneal fibrosis was established as previously described^21^. Briefly, male 7-week-old Sprague-Dawley rats weighing 200–220 g acquired from SLAC Laboratory Animal Co., Ltd. All experiments were conducted according to the Animal Research: Reporting of In Vivo Experiments (ARRIVE) guidelines and were approved by the Laboratory Animal Welfare & Ethics Committee of the Shanghai Pudong New Area People’s Hospital (approval number: 2025-D-78). All methods were performed in accordance with the relevant guidelines and regulations. The rats were housed in a room with a 12-hour light/dark cycle and a temperature maintained at 24 ± 0.5 °C.

The rats were received daily intraperitoneal injections of 20 mL PD fluid containing 4.25% glucose (Baxter Healthcare, USA). Additionally, lipopolysaccharide (LPS; Sigma, L2012) was injected intraperitoneally at a dose of 0.6 mg/kg on days 1, 3, 5, and 7. Control rats were injected with PBS only (*n* = 6). Meanwhile, PD rats were randomly divided into 3 groups, and each group received weekly tail vein injections of either PBS, Exo-C, Exo-M (100 µg/rat^22^, with 6 rats in each group. After 28 days of treatment, the rats were anesthetized​ with pentobarbital sodium (Sigma, 57-33-0, 50 mg/kg, intraperitoneally) before being euthanized​ via cervical dislocation for the collection of peritoneal tissues.

### Histologic evaluation

For histological analysis, the rats were anesthetized, and peritoneal tissues were collected. The tissues were fixed in 4% paraformaldehyde, embedded in paraffin, and sectioned at a thickness of 4 μm. The sections were deparaffinized with xylene and rehydrated through a graded ethanol series. To observe general morphology and peritoneal injury, sections were stained with hematoxylin and eosin (HE; Beyotime, C0105S). Masson’s trichrome staining (Beyotime, C0189S) was performed to evaluate the degree of peritoneal fibrosis, which was quantified by measuring the relative area of blue-stained collagen fibers. After staining, the sections were mounted with neutral balsam and examined under an optical microscope (OLYMPUS, CX21FS1, Tokyo, Japan). The extent of peritoneal injury or fibrosis in each group was analyzed using Image-Pro Plus 6.0 software.

### Statistical analysis

All data were shown as mean ± SD, and all experiments were performed at least three times independently. Differences among groups were analyzed using Student’s t-test or one-way analysis of variance (ANOVA), or two-way ANOVA with SPSS (version 25.0, Chicago, USA). *P* < 0.05 indicates a significant difference among groups.

## Results

### Macrophage-derived CCL24 promotes MMT in PMCs

**Fig. 1:**
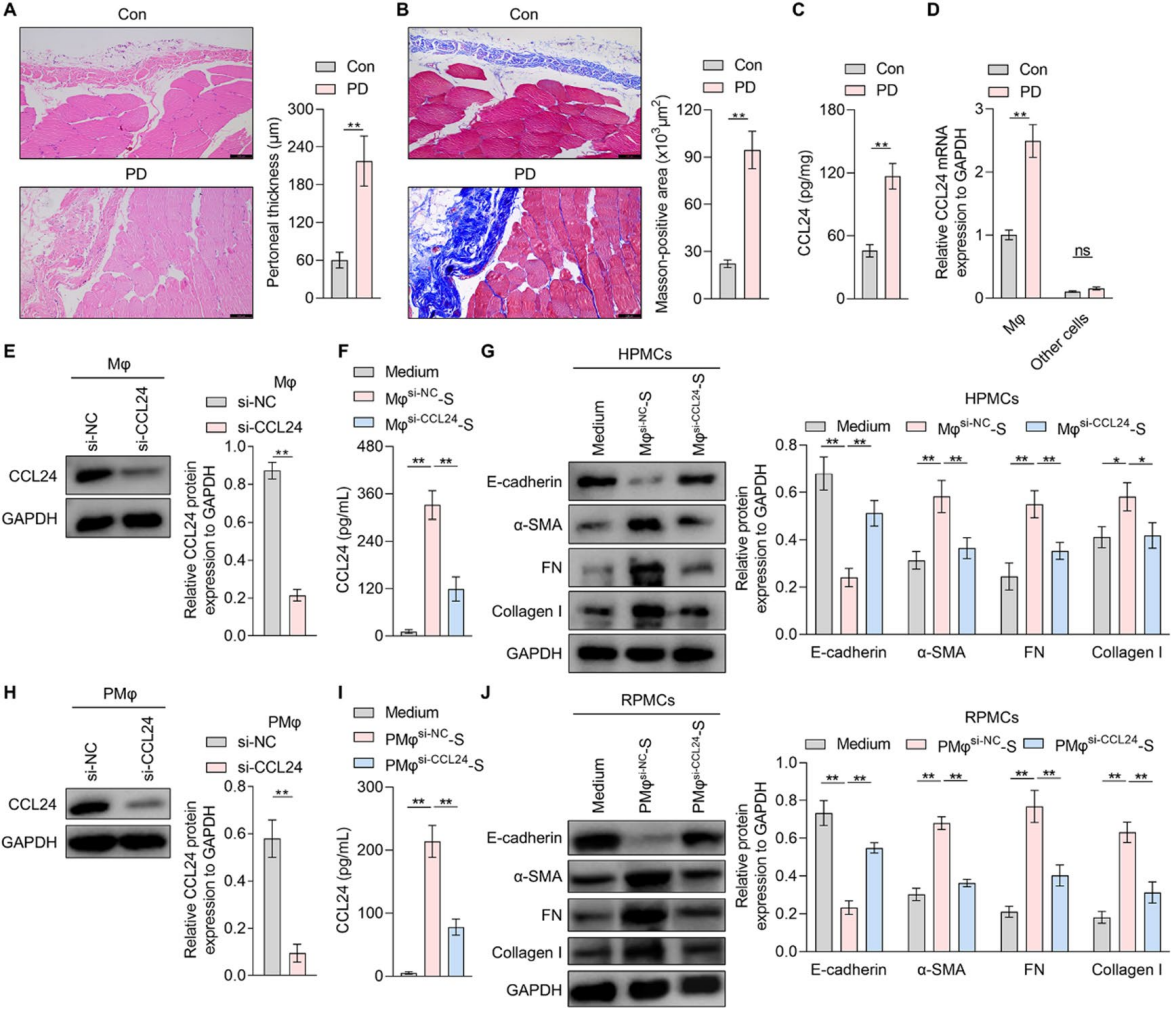
The effects of macrophage-derived CCL24 on MMT in PMCs. A-D. A PD-related peritoneal fibrosis rat model was constructed with intraperitoneal injection of 4.25% glucose PD solution and LPS. Then the peritoneal tissues were collected (*n* = 6/group). (**A**) HE staining was conducted to measure the thickness of the peritoneal membrane in control rats and PD rats (quantified in the right). Scale bars: 100 μm. (**B**) Masson’s trichrome staining of peritoneal tissues from rats of different groups (quantified in the right). Scale bars: 100 μm. (**C**) An ELISA kit was used to detect the abundance of CCL24 in peritoneal tissues. (**D**) Macrophages were obtained from the peritoneal tissues, and then the CCL24 mRNA level in macrophages and other cells in the peritoneal tissues from rats with different treatments was measured by qRT-PCR. (**E**) After differentiation into M0 macrophages with stimulation of phorbol 12-myristate 13-acetate (PMA) for 48 h, THP1 cells were transfected with si-NC or si-CCL24. After being treated with recombinant protein IL-4 (20 ng/mL) and IL-13 (20 ng/mL) for another 48 h, CCL24 protein expression in macrophages was detected with western blotting (quantified in the right). (**F**) ELISA kit was adopted to detect the content of CCL24 in medium or the supernatant of THP-1-derived macrophages exposed to si-NC (Mφ^si−NC^-S) or si-CCL24 (Mφ^si−CCL24^-S). (**G**) Western blotting was used to measure the protein expression of E-cadherin, α-SMA, FN, and collagen I in HPMCs exposed to medium or the supernatant from THP1-derived macrophages with different treatments (quantified in the right). (H) Rat primary peritoneal macrophages (PMφ) were transfected with si-NC or si-CCL24 for 48 h. Then CCL24 protein expression in macrophages was detected with western blotting (quantified in the right). (**I**) ELISA kit was adopted to detect the content of CCL24 in medium or the supernatant of rat primary peritoneal macrophages exposed to si-NC (PMφ^si−NC^-S) or si-CCL24 (PMφ^si−CCL24^-S). (**J**) Western blotting was used to measure the protein expression of E-cadherin, α-SMA, FN, and collagen I in RPMCs exposed to medium or the supernatant of rat primary peritoneal macrophages with different treatments (quantified in the right). Values are the mean ± SD; Student’s t-test (panel: A-E and H) or One-way ANOVA with Tukey’s post hoc test (panel: F-G and I-J); * *p* < 0.05, ** *p* < 0.01, ns *p* > 0.05. PD: peritoneal dialysis; Mφ: THP1-derived macrophages; PMφ: rat primary peritoneal macrophages; MMT: Mesothelial-to-mesenchymal transition; HPMCs: human peritoneal mesothelial cells; FN: fibronectin; RPMCs: rat peritoneal mesothelial cells.

To investigate the role of CCL24 in PD-related fibrosis, a rat model of PD-induced peritoneal injury was established via intraperitoneal injection of 4.25% glucose PD solution and LPS. HE staining indicated marked peritoneal membrane thickening in the PD group compared to controls (Fig. 1A). Masson’s trichrome staining confirmed extensive collagen deposition and ECM accumulation, indicative of progressive fibrosis in PD rats (Fig. 1B), indicating the successful construction of the PD-related fibrosis rat model. Subsequent quantification of peritoneal CCL24 by ELISA showed a significant increase in PD rats compared to controls (Fig. 1C). Interestingly, CCL24 was mainly expressed in peritoneal macrophages rather than other cells in the peritoneal tissue, which was upregulated in the PD rats (Fig. 1D), identifying macrophages as the primary source CCL24 in peritoneal tissues, with significantly elevation in PD-related fibrosis.

Given prior reports linking CCL24 to M2 macrophages^9^, THP1-derived M0 macrophages were polarized to M2 using recombinant proteins IL-4 and IL-13. It was found in flow cytometry and ELISA experiments that M2 markers CD206 and CD163 expression levels were increased in THP1-derived macrophages exposed to IL-4 and IL-13 (Fig.S1A-S1B), accompanied by enhanced secretion of IL-10 (Fig.S1C), confirming the success of M2 polarization. Critically, it was revealed in qRT-PCR assay and western blotting experiments that the mRNA level and protein expression of CCL24 were significantly increased in M2 macrophages (Fig.S1D-S1E), while results from ELISA experiment showed that the content of CCL24 protein was also significantly enhanced in the supernatant from M2 macrophages (Fig.S1F), indicating that M2 macrophages exhibit enhanced capacity for CCL24 synthesize and secrete. To delineate the role of macrophage-derived CCL24 in MMT, we silenced CCL24 in THP1-derived macrophages using siRNA (Fig. 1E), and ELISA confirmed a reduction in CCL24 secretion (Fig. 1F). Then, the supernatant from THP1-derived macrophages was collected to stimulate HPMCs. It was observed in the western blot assay that the supernatant of THP1-derived macrophages with NC siRNA significantly reduced the protein content of E-cadherin, and promoted protein expression of α-SMA and ECM components FN and collagen I in HPMCs (Fig. 1G). However, CCL24 knockdown in THP1-derived macrophages reduced the promoting effects of its supernatant on MMT (Fig. 1G). Moreover, primary peritoneal macrophages, which were isolated from rats and identified by CD68 expression (Fig.S2), also had the ability to inhibit the expression of E-cadherin and promote protein levels of α-SMA, FN and collagen I in RPMCs depending on CCL24 (Fig. 1H and J). It indicates that CCL24 secreted by macrophages directly triggers MMT in HPMCs.

### HpMSCs can improve macrophage-mediated MMT in HPMCs by inhibiting the secretion of CCL24 in macrophages

**Fig. 2:**
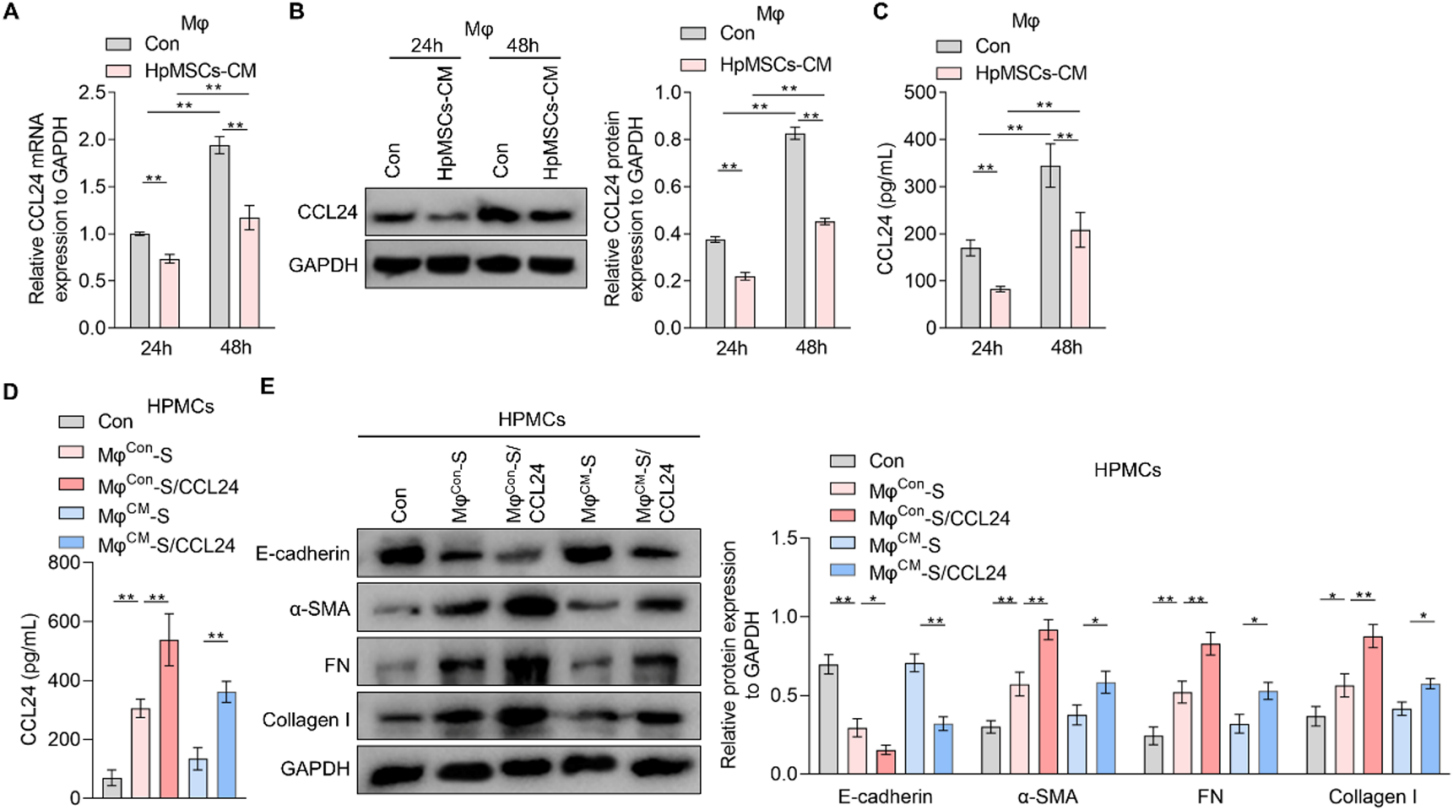
The effects of CCL24 and HpMSCs-CM on macrophage-driven MMT in HPMCs. A-C. THP1-derived macrophages were treated with medium or HpMSCs-derived conditional medium (HpMSCs-CM) together with recombinant protein IL-4 (20 ng/mL) and IL-13 (20 ng/mL) for 24–48 h. (**A**) The mRNA level of CCL24 in THP1-derived macrophages with different treatments was measured by qRT-PCR assay. (**B**) Western blotting was adopted to detect the protein expression of CCL24 in THP1-derived macrophages from different groups (quantified in the right). (**C**) An ELISA kit was used to detect the content of CCL24 in the supernatant of THP1-derived macrophages. (**D**) ELISA kit was used to measure the content of CCL24 in medium or the supernatant of THP1-derived macrophages exposed to medium (Mφ^Con^-S) or HpMSCs-CM (Mφ^CM^-S), together with or without recombinant protein CCL24 (100 ng/mL). (**E**) Western blotting was adopted to measure the protein expression of E-cadherin, α-SMA, FN, and collagen I in HPMCs stimulated with medium or the supernatant of THP1-derived macrophages with different treatments (quantified in the right). Values are the mean ± SD; Two-way ANOVA (Panel: A-C) or One-way ANOVA (Panel: D-E) with Tukey’s post hoc test; * *p* < 0.05, ** *p* < 0.01. MMT: Mesothelial-to-mesenchymal transition; Mφ: THP1-derived macrophages; HPMC: human peritoneal mesothelial cells; HpMSCs: human peritoneal mesenchymal stem cells; FN: fibronectin.

To explore the improvement of HpMSCs on CCL24 synthesis and secretion in macrophages, THP1-derived M2 macrophages were incubated with HpMSCs-CM. qRT-PCR analysis revealed a significant downregulation of CCL24 mRNA levels and protein expression in THP1-derived macrophages exposed to HpMSCs-CM (Fig. 2A and B). Similar results were obtained in the ELISA assay, meaning that the protein content of CCL24 was decreased in the supernatant of THP1-derived macrophages stimulated with HpMSCs-CM (Fig. 2C). These data demonstrated that HpMSCs-CM effectively suppresses CCL24 synthesis and secretion in macrophages. Then, to delineate the functional role of HpMSCs-CM on macrophage-derived CCL24 on MMT, the supernatant from THP1-derived macrophages with or without treatment of HpMSCs-CM was collected to stimulate HPMCs combined with recombinant protein CCL24. ELISA experiments confirmed that the exogenous CCL24 completely abrogated HpMSCs-CM-induced reduction of CCL24 in THP1-derived macrophage supernatants (Fig. 2D). It was also observed that exogenous CCL24 significantly inhibited the expression of E-cadherin and increased protein level of α-SMA, FN, and collagen I in HPMCs (Fig. 2E), confirming the promoting function of CCL24 on the MMT of HPMCs. Meanwhile, it was also found that the promoting effect of the supernatant from THP1-derived macrophages treated with HpMSCs-CM on the MMT in HPMCs was significantly reduced, effect completely rescued by supplementing recombinant protein CCL24 (Fig. 2E). It indicated that HpMSCs-CM can inhibit CCL24 synthesis and secretion in macrophages and then improve MMT in HPMCs.

### The inhibitory effects of HpMSCs on macrophage-mediated MMT in HPMCs depend on the CCR3/p38 pathway

**Fig. 3:**
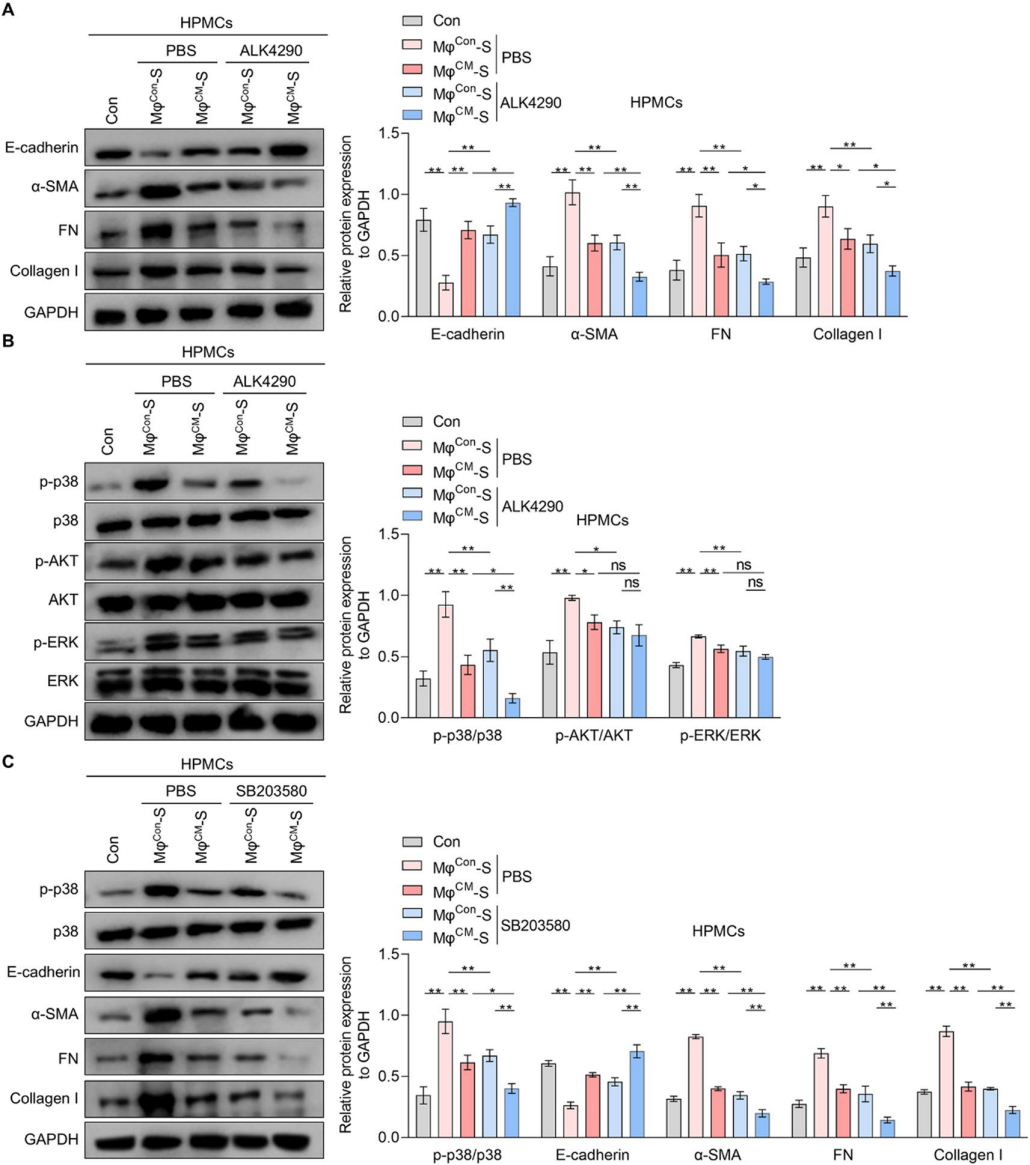
The role of CCR3 and the MAPK pathway in the inhibitory effects of HpMSCs-CM on macrophage-driven MMT in HPMCs. (**A**-**B**) After pre-treatment of CCR3 inhibitor ALK4290 (5 µM) for 1 h, HPMCs were exposed to medium or the supernatant from THP1-derived macrophages treated with medium (Mφ^Con^-S) or HpMSCs-conditional medium (Mφ^CM^-S). Then, western blotting was used to detect the protein expression of E-cadherin, α-SMA, FN, and collagen I (A, quantified in the right) and PI3K/AKT and MAPK pathways (B, quantified in the right) in HPMCs. (**C**). After pre-treated with P38 inhibitor SB203580 (10 µM) for 1 h, HPMCs were incubated with medium or macrophage-derived supernatant. Then the phosphorylation level of P38 and the protein level of E-cadherin, α-SMA, FN, and collagen I in HPMCs were measured by western blotting (quantified in the right). Values are the mean ± SD; One-way ANOVA with Tukey’s post hoc test; * *p* < 0.05, ** *p* < 0.01, ns *p* > 0.05. MMT: Mesothelial-to-mesenchymal transition; Mφ: THP1-derived macrophages; HPMC: human peritoneal mesothelial cells; HpMSCs: human peritoneal mesenchymal stem cells; FN: fibronectin.

Prior reports revealed CCR3 as the primary receptor for CCL24^23^. To investigate the dependence of macrophage-derived CCL24-mediated MMT on CCR3, HPMCs were treated with conditional medium from THP1-derived macrophages and the CCR3-specific inhibitor ALK4290. It was observed in the WB experiment that ALK4290 not only abrogated the promoting effect of control macrophage-derived supernatant on the MMT in HPMCs, but also blocked MMT in HPMCs exposed to HpMSCs-CM-treated macrophage-derived supernatant (Fig. 3A), confirming that the regulatory functions of macrophage supernatant and HpMSCs-CM both depend on CCR3. Given the established link between CCR3 and MAPK pathways, including ERK, PI3K/AKT, and P38^24,25^, the phosphorylation levels of P38, ERK, and AKT in HPMCs with different treatments were measured by western blotting. It was found that control supernatant from THP1-derived macrophages induced robust phosphorylation of P38, ERK, and AKT, while the promoting effects of HpMSCs-CM-treated THP1-derived macrophage supernatant on the MAPK pathway were significantly reduced (Fig. 3B). Meanwhile, ALK4290 decreased the phosphorylation level of p38 in HPMCs exposed to supernatant from THP1-derived macrophages treated with or without HpMSCs-CM. To dissect the contribution of p38 signaling, HPMCs were treated with THP1-derived macrophage supernatant together with P38-specific inhibitor SB203580. The results showed that SB203580 significantly reduced p38 phosphorylation and MMT in HPMCs and enhanced the anti-MMT effects of HpMSCs-CM (Fig. 3C). These data indicate that macrophage-derived CCL24 drives MMT via the CCR3/p38 MAPK pathway, while HpMSCs-CM interrupts CCL24 secretion by suppressing this pathway.

### HpMSCs-Exo alleviate macrophage-derived CCL24 synthesis and secretion

**Fig. 4:**
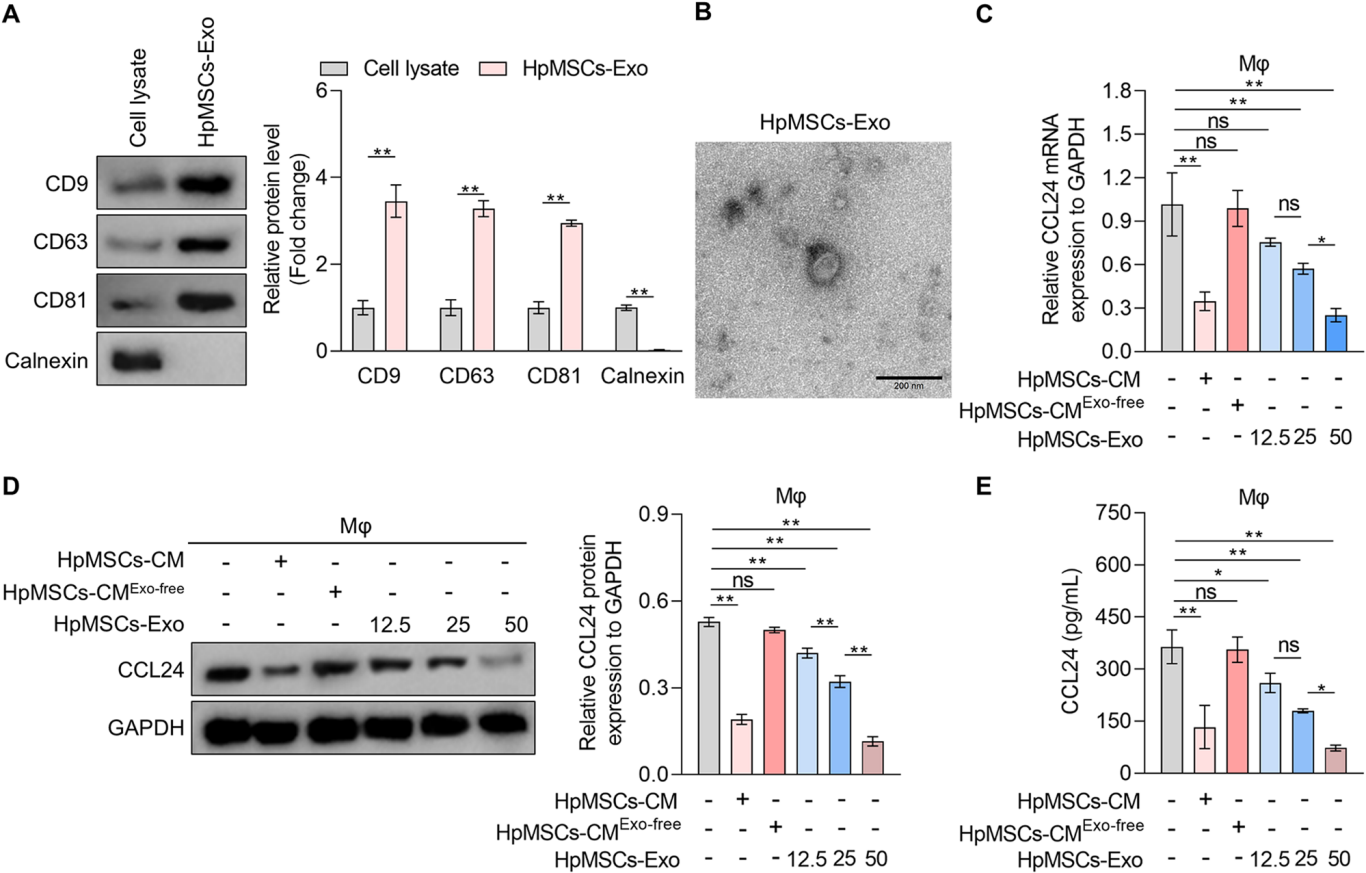
The effects of HpMSCs-Exo on CCL24 synthesis and secretion in macrophages. (**A**-**B**). The exosomes in the supernatant of HpMSCs (HpMSCs-Exo) were collected. (**A**) Western blotting was adopted to measure the protein expression of CD9, CD63, CD81, and Calnexin in exosomes and cell lysis (quantified in the right). (**B**) The morphology of HpMSCs-Exo was observed under a transmission electron microscope. Scale bars: 200 nm. C-E. THP1-derived macrophages were incubated with HpMSCs-conditional medium (HpMSCs-CM) or exosome-free HpMSCs-CM (HpMSCs-CM^Exo−free^) or different concentrations of HpMSCs-Exo (12.5, 25, 50 µg/mL). qRT-PCR (**C**) and western blotting (**D**) were used to measure the mRNA level and protein expression of CCL24 in macrophages, and an ELISA kit was adopted to detect the protein content of CCL24 in the supernatant from macrophages (**E**). Values are the mean ± SD; Student’s t-test (Panel: A) or One-way ANOVA with Tukey’s post hoc test (Panel: C-E); * *p* < 0.05, ** *p* < 0.01, ns *p* > 0.05. Mφ: THP1-derived macrophages; HpMSCs: human peritoneal mesenchymal stem cells.

Given the critical role of exosomes in MSCs-mediated intercellular communication and the therapeutic potential of exosomes^26^, exosomes were isolated from HpMSCs. And then western blot assay confirmed the enrichment of exosomal biomarkers^27^ CD9, CD63, and CD81 in HpMSCs-Exo, with minimal expression in corresponding cell lysates (Fig. 4A). In contrast, calnexin (an endoplasmic reticulum chaperone^28^ was exclusively detected in cell lysis, serving as a negative control for exosomes identification. Moreover, the transmission electron microscope revealed spherical vesicles with a characteristic cup-shaped morphology and a mean diameter of approximately 100 nm (Fig. 4B), validating the successful isolation of exosomes. To delineate the functional contribution of HpMSCs-Exo, exosomes were removed from HpMSCs-CM via high-speed centrifugation to generate exosome-free HpMSCs-CM (HpMSCs-CM^Exo−free^), and then treated with macrophages. The results of the qRT-PCR and western blotting experiments revealed no significant inhibitory effects of HpMSCs-CM^Exo−free^ on CCL24 mRNA level and protein expression compared to HpMSCs-Exo (Fig. 4C and D). Secreted CCL24 levels in supernatant from THP1-derived macrophages treated with HpMSCs-CM^Exo−free^ reverted to baseline (Fig. 4E). More importantly, HpMSCs-Exo alone recapitulated the anti-CCL24 synthesis and secretion effects of intact HpMSCs-CM in a dose-dependent manner (Fig. 4C and E). It indicates that HpMSCs primarily suppress CCL24 synthesis and secretion in macrophages via exosomes, independent of soluble factors in conditioned medium.

### HpMSCs-Exo deliver miR-320d to macrophages leading to KLF7/STAT3/CCL24 pathway inhibition

**Fig. 5:**
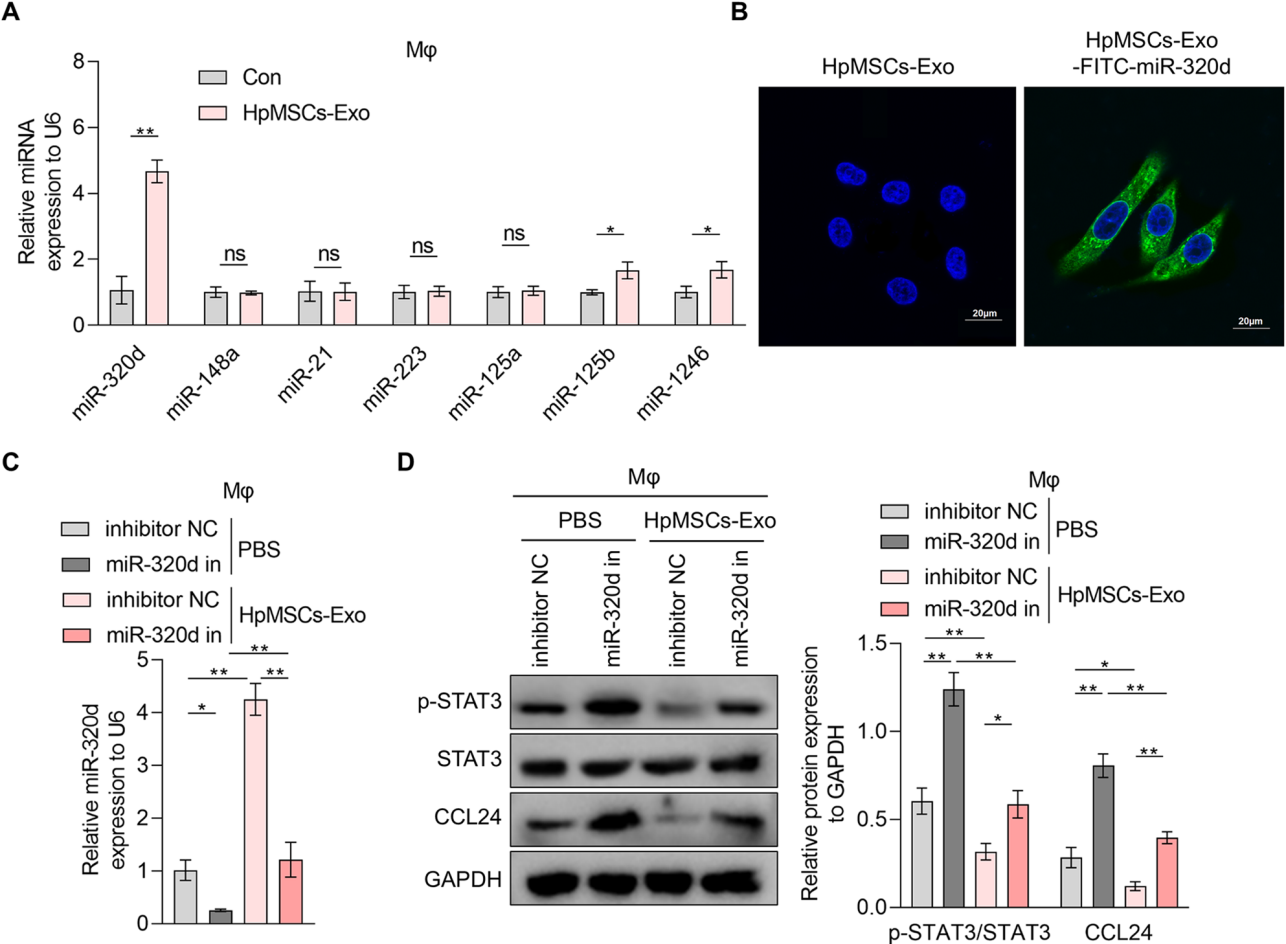
The effects of HpMSCs-Exo and miR-320d on CCL24 expression in macrophages. (**A**) The miRNA level in THP1-derived macrophages treated with or without HpMSCs-derived exosomes (HpMSCs-Exo) was measured with qRT-PCR assay. (**B**) After incubating with HpMSCs-Exo with or without FITC-labeled miR-320d, FITC in macrophages was observed under a fluorescence microscope. Scale bars: 20 μm. C-D. THP1-derived macrophages were treated with or without HpMSCs-Exo together with miRNA inhibitor NC (inhibitor NC) or miR-320d inhibitor (miR-320d in). (**C**) qRT-PCR assay was conducted to detect the level of miR-320d in macrophages. (**D**) The protein levels of p-STAT3, STAT3, and CCL24 in macrophages with different treatments were measured with western blotting (quantified in the right). Values are the mean ± SD; Student’s t-test (Panel: A) or One-way ANOVA with Tukey’s post hoc test (Panel: C-D); * *p* < 0.05, ** *p* < 0.01, ns *p* > 0.05. Mφ: THP1-derived macrophages; HpMSCs: human peritoneal mesenchymal stem cells.

Prior study reports that STAT3 binding to the CCL24 promoter drives its transcriptional activation in clear-cell renal cell carcinoma^23^. To investigate whether HpMSCs-Exo modulate this interaction, ChIP-qPCR experiments were performed. Results demonstrated significant enrichment of STAT3 in the CCL24 promoter region in THP1-derived macrophages, which was alleviated by HpMSCs-Exo (Fig.S3), indicating that HpMSCs-Exo disrupts STAT3-mediated transcriptional regulation of CCL24. Based on literature screening of miRNAs in MSCs-Exo implicated in STAT3 pathway suppression, we identified seven candidates (miR-148a^29^, miR-21^30^, miR-223^31^, miR-125a^32^, miR-125b^32^, miR-1246^33^ and miR-320d^33^. qRT-PCR analysis revealed that HpMSCs-Exo significantly upregulated miR-320d, miR-125b, and miR-1246 in THP1-derived macrophages, with miR-320d showing the most pronounced induction (Fig. 5A). Next, IF experiment confirmed THP1-derived macrophages uptake of FITC-labeled miR-320d delivered by HpMSCs-Exo (Fig. 5B). Furthermore, it was observed in qRT-PCR assay that the abundance of miR-320d in HpMSCs-Exo was much higher than the increase amount of miR-320d in THP1-derived macrophages after treatment with HpMSCs-Exo (Fig.S4), ruling out the possibility that the increase is through induction. Then, macrophages were co-treated with miR-320d inhibitor combined with HpMSCs-Exo. qRT-PCR analysis showed that miR-320d inhibitor not only significantly reduced the abundance of miR-320d in THP1-derived macrophages, but also abolished HpMSCs-Exo-induced miR-320d up-regulation (Fig. 5C). WB assay results showed that miR-320d inhibitor increased p-STAT3 level and CCL24 protein expression in THP1-derived macrophages, and reversed the inhibitory effect of HpMSCs-Exo on the levels of p-STAT3 and CCL24 protein (Fig. 5D). However, miR-320d inhibitor and HpMSCs-Exo both had no significant effects on the protein expression of total STAT3 in THP1-derived macrophages. It indicates that HpMSCs-Exo delivers miR-320d to macrophages, suppressing STAT3 phosphorylation and CCL24 synthesis in macrophages without altering total STAT3 expression.

**Fig. 6:**
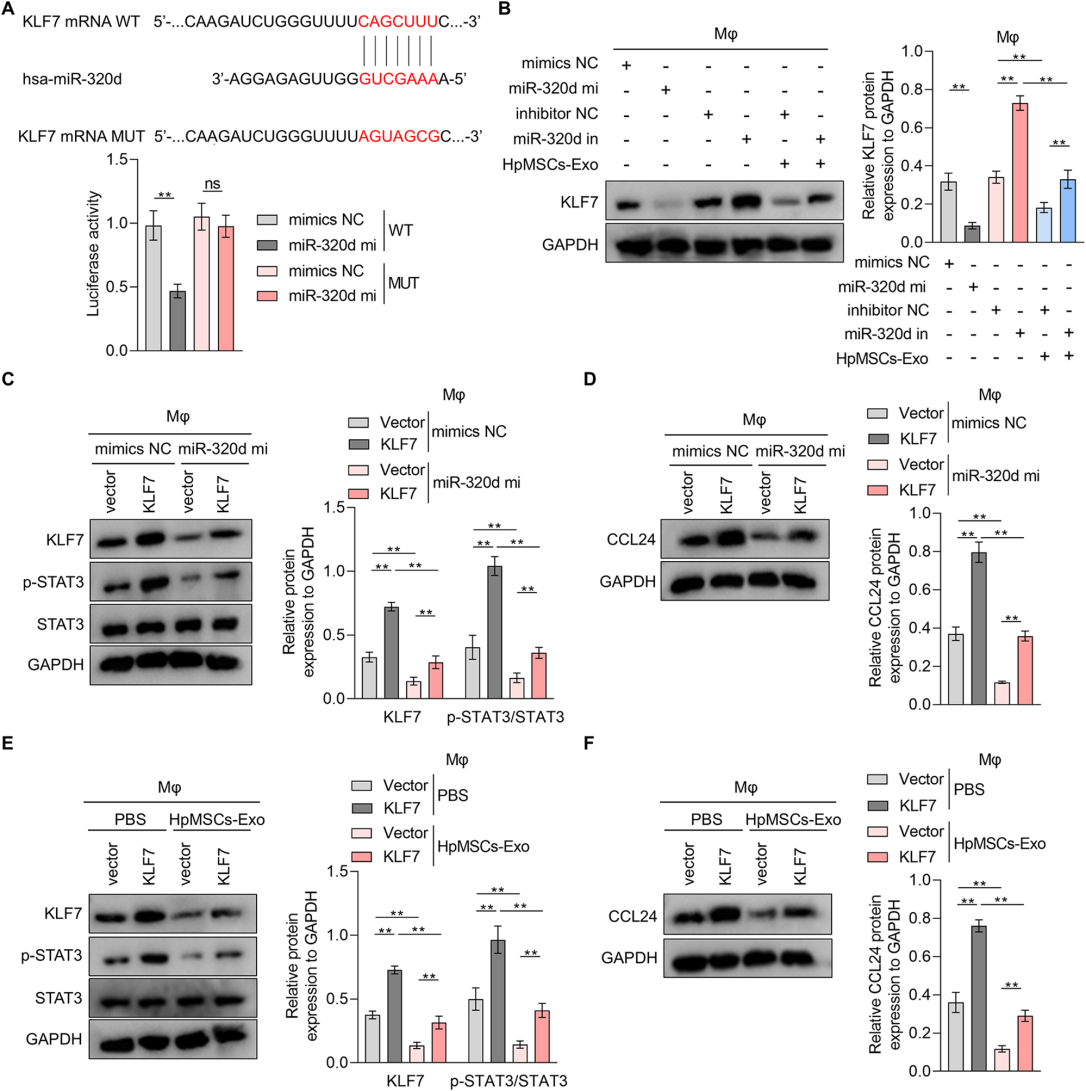
The effects of KLF7 on HpMSCs-Exo or miR-320d-mediated STAT3/CCL24 pathway inhibition in macrophages. (**A**). After transfection with luciferase reporter plasmids containing wild or mutated KLF7 mRNA 3’ UTR sequence (displayed in the left), which binds to miR-320d in TargetScan, THP1-derived macrophages were treated with miRNA mimics NC (mimics NC) or miR-320d mimics (miR-320d mi). Then the luciferase activity in macrophages was measured. (**B**). Western blotting was conducted to measure the protein expression of KLF7 in macrophages treated with or without HpMSCs-derived exosomes (HpMSCs-Exo) in the presence of miR-320d mimics or miR-320d inhibitor (quantified in the right). (**C**-**D**). THP1-derived macrophages were transfected with miRNA mimics NC or miR-320d mimics together with vector or KLF7 overexpression plasmids. Western blotting was adopted to detect the protein expression of KLF7 and the phosphorylation level of STAT3 (**C**) and the protein level of CCL24 (**D**) in macrophages. E-F. After being transfected with vectors or KLF7 overexpression plasmids, THP1-derived macrophages were incubated with or without HpMSCs-Exo. Western blotting was used to measure the protein expression of KLF7 and the phosphorylation level of STAT3 (**E**) and the protein level of CCL24 (**F**) in macrophages. Values are the mean ± SD; Student’s t-test (Panel: A) or One-way ANOVA with Tukey’s post hoc test (Panel: B-F); * *p* < 0.05, ** *p* < 0.01, ns *p* > 0.05. Mφ: THP1-derived macrophages; HpMSCs: human peritoneal mesenchymal stem cells.

Bioinformatic prediction (TargetScan) identified a conserved binding site for miR-320d in the 3’UTR terminal of KLF7 mRNA (Fig. 6A). Functional validation was performed by luciferase reporter assay, and it was observed that miR-320d mimics significantly reduced luciferase activity in THP1-derived macrophages transfected with wild-type KLF7 3’UTR, but not mutant constructs (Fig. 6A). Besides, western blotting assay revealed that miR-320d mimics decreased KLF7 expression, while miR-320d inhibitors triggered KLF7 upregulation (Fig. 6B). HpMSCs-Exo reduced KLF7 expression, an effect reversed by miR-320d inhibitor (Fig. 6B). It indicated that KLF7 is a target of HpMSCs-derived exosomal miR-320d. Furthermore, KLF7 overexpression not only increased STAT3 phosphorylation level and CCL24 expression, but also abrogated miR-320d mimics-induced suppression of KLF7, p-STAT3, and CCL24 in THP1-derived macrophages (Fig. 6C and D). Moreover, KLF7 overexpression reversed HpMSCs-Exo-mediated downregulation of KLF7, p-STAT3 and CCL24 in THP1-derived macrophages (Fig. 6E and F). These data indicate that HpMSCs-Exo inhibits miR-320d-dependent KLF7/STAT3 pathway in macrophages, contributing to a reduction of CCL24 synthesis.

### HpMSCs-Exo loaded with miR-320d inhibit CCL24 secretion in macrophages, MMT of PMCs and the progression of PD-related fibrosis

**Fig. 7:**
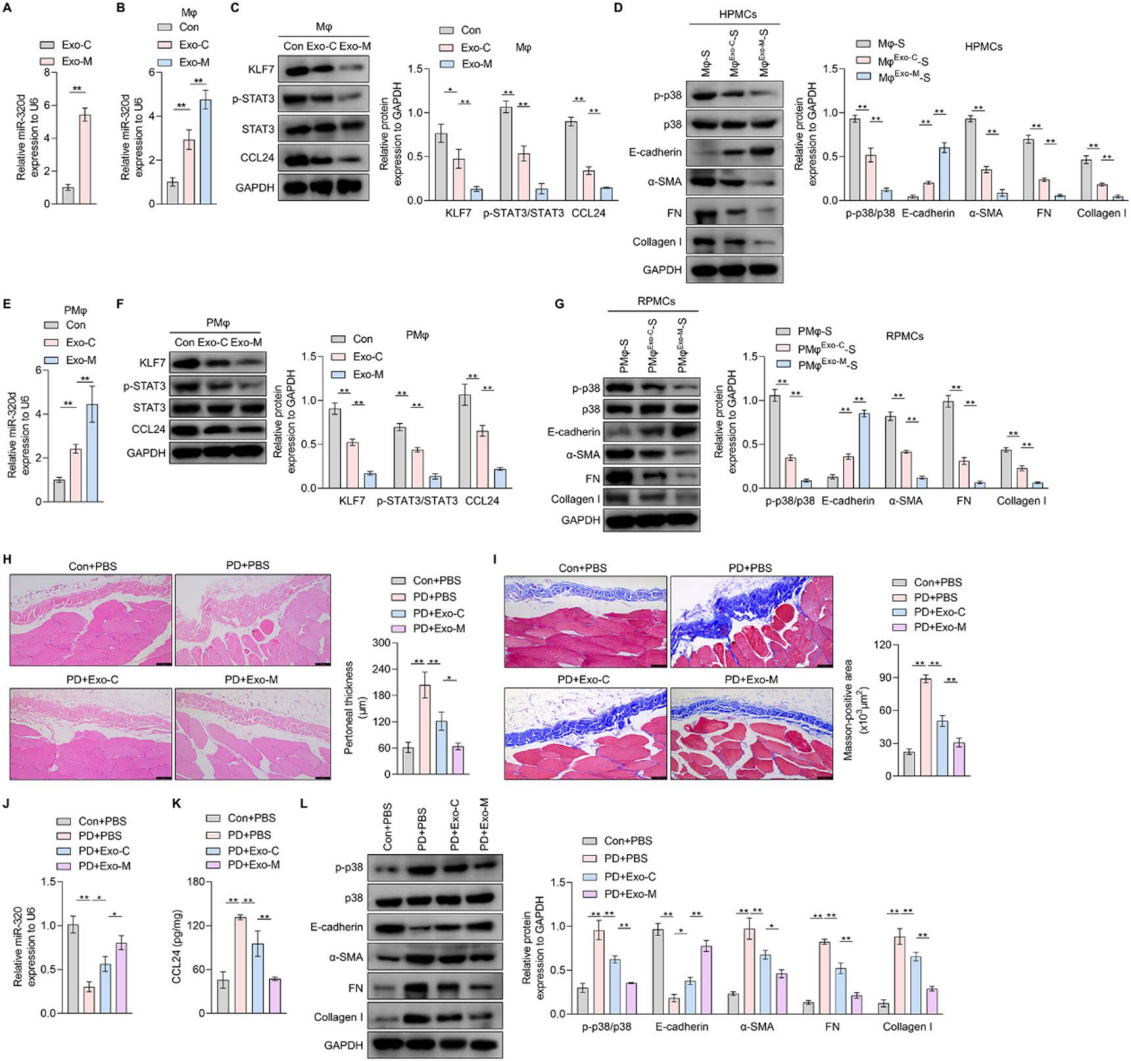
The effects of modified HpMSCs-Exo loaded with miR-320d on KLF7/STAT3/CCL24 pathway, MMT, and PD-related fibrosis. (**A**). Exosomes were separated from the supernatant of HpMSCs transfected with miRNA mimics NC or miR-320d mimics, and then the abundance of miR-320d in native exosomes (Exo-C) and modified exosomes (Exo-M) was measured with qRT-PCR assay. B-C. THP1-derived macrophages were incubated with Exo-C or Exo-M. And then qRT-PCR (**B**) and western blotting (**C**) were adopted to measure the level of miR-320d and the protein expression of KLF7 and CCL24, and the phosphorylation level of STAT3 (quantified in the right). (**D**). Western blotting was used to measure the phosphorylation level of p38 and the protein content of E-cadherin, α-SMA, FN, and collagen I in HPMCs treated with the supernatant of THP1-derived macrophages stimulated with medium (Mφ-S) or Exo-C (Mφ^Exo−C^-S), or Exo-M (Mφ^Exo−M^-S) (quantified in the right). (**E**-**F**). Rat primary peritoneal macrophages were incubated with Exo-C or Exo-M. And then qRT-PCR (**E**) and western blotting (**F**) were adopted to measure the level of miR-320d and the protein expression of KLF7 and CCL24, and the phosphorylation level of STAT3 (quantified in the right). (**G**). Western blotting was used to measure the phosphorylation level of p38 and the protein content of E-cadherin, α-SMA, FN, and collagen I in RPMCs treated with the supernatant of rat primary peritoneal macrophages stimulated with medium (PMφ-S) or Exo-C (PMφ^Exo−C^-S), or Exo-M (PMφ^Exo−M^-S) (quantified in the right). (**H**-**L**). A PD-associated peritoneal injury rat model was established with intraperitoneal injection of 4.25% glucose PD solution and LPS. And then PD rats were divided into three groups and subjected to different treatments, including tail vein injection of PBS or Exo-C or Exo-M (*n* = 6/groups). Meanwhile, 6 control rats were only exposed to intraperitoneal and tail vein injection of PBS. Then the peritoneal tissues were collected. (**H**). HE staining was conducted to measure the thickness of the peritoneal membrane in control rats and PD rats with different treatments (quantified in the right). Scale bars: 100 μm. (**I**). Masson staining of peritoneal tissues from rats of different groups. Scale bars: 100 μm. (**J**). The level of miR-320d in the peritoneal tissues from different rats was detected by qRT-PCR. (**K**). ELISA kit was used to measure the content of CCL24 in peritoneal tissues. (**L**). Western blotting was conducted to detect the phosphorylation level of p38 and the protein content of E-cadherin, α-SMA, FN, and collagen I in the peritoneal tissues (quantified in the right). Values are the mean ± SD; Student’s t-test (Panel: A) or One-way ANOVA with Tukey’s post hoc test (Panel: B-L); * *p* < 0.05, ** *p* < 0.01, ns *p* > 0.05. Mφ: THP1-derived macrophages; PMφ: Rat primary peritoneal macrophages; MMT: Mesothelial-to-mesenchymal transition; HpMSCs: human peritoneal mesenchymal stem cells; HPMC: human peritoneal mesothelial cells; FN: fibronectin; PD: peritoneal dialysis.

To potentiate the inhibitory effect of HpMSCs-Exo on CCL24 synthesis in macrophages, modified miR-320d-enriched HpMSCs-Exo (Exo-M) was constructed. Successful miR-320d loading in HpMSCs-Exo was by qRT-PCR assay (Fig. 7A). When co-cultured with THP1-derived macrophages, Exo-M induced higher miR-320d abundance than control HpMSCs-Exo (Exo-C) (Fig. 7B), demonstrating efficient cellular delivery of exosomal miR-320d. Moreover, compared to Exo-C, Exo-M exhibited enhanced inhibitory effects on KLF7 expression, STAT3 phosphorylation, and CCL24 abundance in THP1-derived macrophages (Fig. 7C). More importantly, compared to HPMCs treated with control THP1-derived macrophage supernatant, p-p38 level and MMT were suppressed in those exposed to the supernatant from HpMSCs-Exo-treated THP1-derived macrophages, especially Exo-M (Fig. 7D). Furthermore, Exo-C and Exo-M both increased the abundance of miR-320d, and inhibited KLF7 expression, STAT3 phosphorylation, and CCL24 content in rat primary peritoneal macrophages, the latter owning stronger effects (Fig. 7E and F). Meanwhile, compared to control macrophage supernatant-treated RPMCs, p38 pathway activation level and MMT was also inhibited in RPMCs treated with HpMSCs-Exo-exposed macrophage-drived supernatant, with Exo-M exhibiting stronger regulatory effects (Fig. 7G). It demonstrated that modified HpMSCs-Exo loaded with miR-320d outperform native exosomes in blocking macrophage-driven MMT through improving miR-320d/KLF7/STAT3/CCL24/p38 pathway.

Subsequently, PD-related peritoneal fibrosis rats were administered either Exo-C or Exo-M to investigate the therapeutic potential of HpMSCs-Exo on peritoneal fibrosis associated with PD. HE staining revealed that both types of HpMSCs-Exo attenuated peritoneal tissue thickening in PD rats, with Exo-M exhibiting a more pronounced inhibitory effect (Fig. 7H). Consistent with this, Masson’s trichrome staining demonstrated amelioration of peritoneal fibrosis in PD rats following treatment of either Exo-C or Exo-M, the latter showing superior efficacy (Fig. 7I). Quantitative RT-PCR analysis indicated a significant downregulation of miR-320d in the peritoneal tissue of PD rats, which was rescued by HpMSCs-Exo treatment (Fig. 7J). Notably, Exo-M induced a more substantial restoration of miR-320d abundance (Fig. 7J). ELISA results further revealed that upregulation of CCL24 in the peritoneal tissues of PD rats was markedly suppressed by HpMSCs-Exo, with Exo-M exerting a stronger inhibitory effect (Fig. 7K). In line with the CCL24 findings, western blotting analysis showed elevated phosphorylation levels of p38 and increased protein expression of α-SMA, FN and collagen I, accompanied by reduced E-Cadherin expression in PD rats, all of which were counteracted by HpMSCs-Exo treatment (Fig. 7L). Importantly, Exo-M produced more potent inhibition of p38 phosphorylation and MMT compared to Exo-C (Fig. 7L). These results collectively suggest that HpMSCs-Exo, especially modified HpMSCs-Exo loaded with miR-320d, alleviate PD-associated peritoneal fibrosis via the miR-320d/KLF7/STAT3/CCL24/P38 signaling pathway.

## Discussion

Previous studies have reported that CCL24 contributes to fibrosis in the skin and lung, and that such fibrotic processes can be attenuated by a blocking monoclonal antibody against CCL24^34^. Although an increase in Macro4 macrophages expressing high levels has been observed in PD rats^8^, the functional role of CCL24 in PD-associated fibrosis remained unclear. Here, we demonstrated for the first time that CCL24 expression is upregulated in the peritoneal tissue of PD rats. Moreover, exogenous administration of recombinant CCL24 promoted MMT in HPMCs, indicating its active involvement in PD-related fibrogenesis. These findings suggest that neutralization of CCL24 using a specific antibody may represent a potential therapeutic strategy to ameliorate peritoneal fibrosis in PD, though further validation is required. It is known that macrophages can produce CCL24^9^, which was corroborated in our experimental setting. The receptor for CCL24, CCR3, has been shown to activate the MAPK signaling pathway in trophoblasts and neutrophils^24,25^. Similarly, we found that treatment of HPMCs with CCL24 induced p-p38 upregulation, implying that CCL24 triggers a CCR3-dependent p38 pathway activation that drives MMT. Therefore, targeted inhibition of the CCL24/CCR3/P38 axis may offer a novel therapeutic approach for mitigating PD-related fibrosis, a hypothesis that warrants further experimental confirmation.

Studies have shown that CCL24 upregulation in murine models of allergic rhinitis and airway allergic inflammation can be suppressed by treatment with human embryonic stem cell-derived MSCs^35^ and tonsil-derived MSCs^36^. Additionally, MSCs-Exo have been reported to exert anti-fibrotic effects in the liver^29^. In the present study, we demonstrated that HpMSCs and their exosomes inhibit both the synthesis and secretion of CCL24 in macrophages and subsequent CCL24-mediated MMT in PMCs. Importantly, administration of HpMSCs-Exo alleviated peritoneal fibrosis in a PD rat model, supporting their therapeutic potential in PD-related fibrosis progression. Notably, pMSCs can be isolated from PD effluent, suggesting that this waste fluid may serve as an accessible source of autologous MSCs, thereby offering a promising strategy for clinical management of PD-associated fibrosis.

Exosomal miRNAs are known to contribute significantly to the therapeutic effects of MSCs^14^. Upon uptake by recipient cells, exosomes transfer their miRNA cargo, enabling functional regulation in target tissues. For instance, MSCs can deliver miR-148a via exosomes to macrophages, leading to suppression of the KLF6/STAT3 pathway^29^. In this study, we found that HpMSCs-Exo deliver miR-320d into macrophages, elevating intracellular miR-320d levels, which in turn reduces CCL24 production and secretion, ultimately ameliorating MMT and peritoneal fibrosis. Moreover, engineered MSCs-Exo loaded with specific miRNAs have been shown to enhance therapeutic efficacy compared to native MSCs-Exo^37^. For instance, exosomes derived from miR-146a-5p-overexpressing MSCs exhibit stronger inhibitory effects on microglia pyroptosis^14^. Consistently, we successfully generated miR-320d-enriched HpMSCs-Exo, which exerted more potent suppression on macrophage CCL24 release and MMT in PMCs, and demonstrated superior inhibitory effects on PD-related fibrosis. These findings reinforce the potential of miRNA-loaded MSC exosomes as a therapeutic strategy for fibrotic disease and highlight pMSCs-Exo overexpressing miR-320d as a promising candidate for treating PD-related fibrosis, warranting further validation.

STAT3 has been identified as a key transcriptional regulator that binds to the CCL24 promoter and drives its expression in clear-cell renal cell carcinoma^23^. In line with this, our study demonstrated that HpMSCs-Exo suppress the recruitment of STAT3 to the CCL24 promoter in macrophages, underscoring the dependence of CCL24 downregulation on the STAT3 pathway in this cell type. Notably, STAT3 signaling can be modulated by miR-320d through indirect mechanisms. For example, in vascular endothelial cells, it suppresses GNAI1 expression, thereby indirectly attenuating the JAK2/STAT3 pathway activation without altering STAT3 expression^38^. In macrophages, however, we observed that neither HpMSCs-Exo nor miR-320d affected STAT3 expression. Instead, both HpMSCs-Exo and miR-320d significantly reduced KLF7 expression, suggesting KLF7 as a novel downstream target of miR-320d in this context. Previous study indicates that KLF7 activates the JAK2/STAT3 pathway in the hippocampus following traumatic brain injury^39^. And KLF7 also can bind to the promoter region of PDGFB and enhance its transcriptional activity, and then secreted PDGFB activates JAK/STAT3 signaling pathway through its receptor PDGFR, leading to colon adenocarcinoma progression^40^. Our findings extend this role to macrophages, revealing that KLF7 promotes the STAT3 pathway activation and that HpMSCs-Exo-derived miR-320d suppresses KLF7/STAT3 signaling, ultimately leading to reduced CCL24 synthesis and secretion. Interestingly, STAT3-induced long noncoding RNA LINC00668 has been shown to upregulate KLF7 by sponging miR-193a in non-small cell lung cancer^41^. And miR-193a exerts anti-fibrotic effects in hepatic fibrosis models^42^. Therefore, we speculate that a positive feedback loop may exist between STAT3 and KLF7 in macrophages, potentially amplifying CCL24 production and accelerating PD-associated peritoneal fibrosis. This hypothesis warrants further experimental validation.

In summary, this study demonstrates that HpMSCs-Exo, particularly when loaded with miR-320d, alleviated macrophage-derived CCL24 secretion, CCL24-driven MMT, and PD-associated fibrosis via the miR-320d/KLF7/STAT3 axis. Nevertheless, a limitation of this work is the absence of clinical peritoneal tissue samples from patients undergoing PD treatment to directly correlate the miR-320d/KLF7/STAT3/CCL24 signaling with PD-associated fibrosis progression. Further studies will focus on validating this axis in human samples. Despite this, the consistent findings across cellular and animal models provide robust evidence supporting the critical role of macrophage-derived CCL24 in peritoneal fibrosis and highlight the therapeutic potential of engineered HpMSCs-Exo delivering miR-320d. However, the unclear direct molecular mechanism by which KLF7 regulates STAT3 activation was a mechanistic limitation of the current study, which needs to be revealed in the future.

## Conclusion

**Fig. 8:**
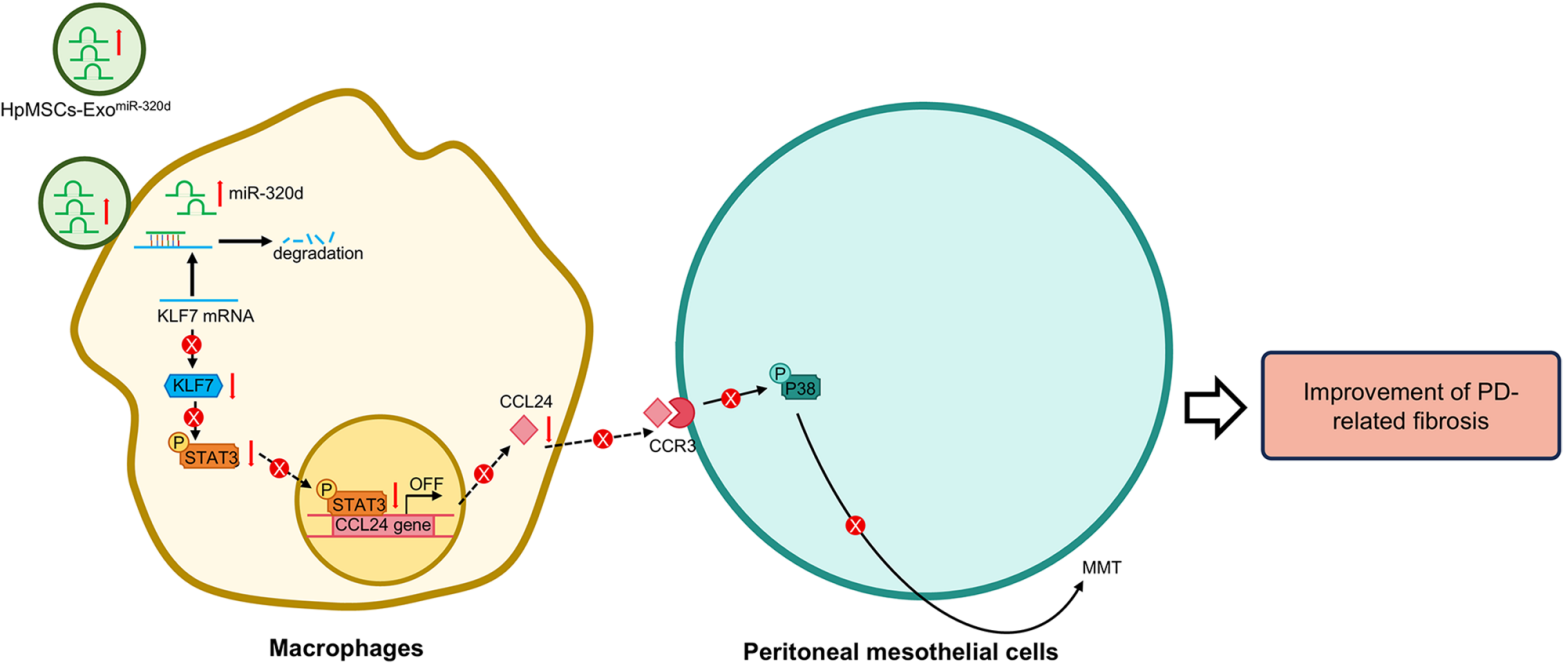
The mechanism diagram by which native or modified HpMSCs-Exo alleviate PD-related fibrosis progression. HpMSCs-Exo, especially modified HpMSCs-Exo loaded with miR-320d, can deliver miR-320d into macrophages, resulting in the upregulation of intracellular miR-320d levels. The enriched miR-320d subsequently binds to KLF7 mRNA and suppresses its protein expression, thereby inhibiting KLF7-dependent STAT3 activation and downstream CCL24 synthesis in macrophages. Reduced secretion of CCL24 attenuates the CCR3/p38 MAPK pathway activation and ameliorates MMT, ultimately alleviating PD-associated fibrosis.

Our findings demonstrate that HpMSCs-Exo deliver miR-320d into macrophages, upregulating its intracellular expression and subsequently suppressing KLF7-mediated STAT3 activation. This cascade ultimately inhibits CCL24 synthesis and secretion, thereby attenuating MMT and ameliorating PD-related fibrosis (Fig. 8). Importantly, HpMSCs-Exo loaded with exogenous miR-320d exhibit enhanced efficacy in blocking this pathway compared to native exosomes. These results highlight the therapeutic potential of targeting the.

miR-320d/KLF7/STAT3/CCL24 axis in PD-related fibrosis. Furthermore, the use of autologous peritoneal mesenchymal stem cell-derived exosomes (pMSCs-Exo) engineered with miR-320d may represent a novel and clinically feasible strategy for mitigating fibrotic progression in PD patients.

## Supplementary Information

Below is the link to the electronic supplementary material.

Supplementary Material 1

Supplementary Material 2

## Data Availability

The data that support the findings of this study are available from the corresponding author upon reasonable request.
