# Supplementary material for: Peritoneal MSCs-derived exosomes suppress CCL24 synthesis through miR-320d delivery contributing to the improvement of peritoneal dialysis-associated fibrosis
Source: Sci Rep. 2026 Mar 4;16:11998. doi: 10.1038/s41598-026-42489-w (PMC13069033; doi:10.1038/s41598-026-42489-w)
Supplement: Supplementary file 1 — Supplementary Material 1 [file 41598_2026_42489_MOESM1_ESM.pdf]

Supplementary figures

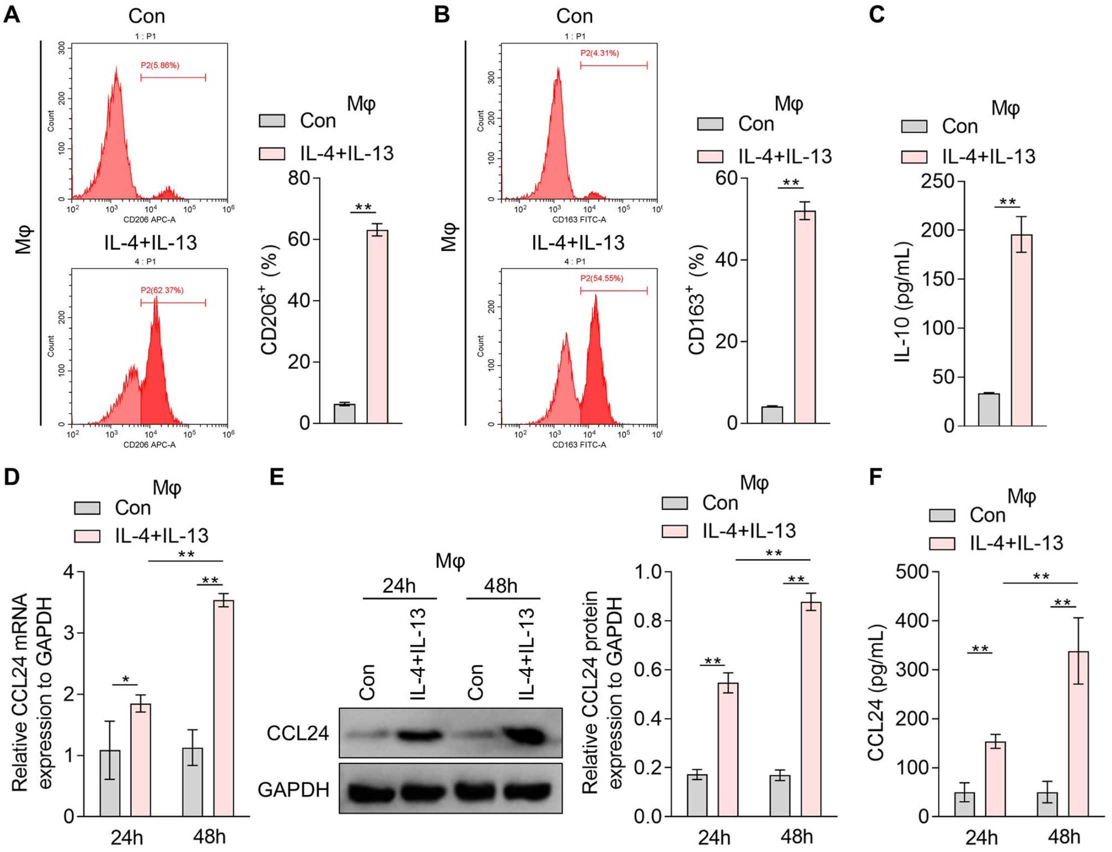

Figure S1. Establishment of CCL24-enriched THP1-derived macrophage model. THP1-derived macrophages were treated with or without recombinant protein IL-4 (20 ng/mL) and IL-13 (20 ng/mL) for 24 h or 48 h. A-B. The distribution of CD206 (A) and CD163 (B) on the cell membrane of macrophages was detected by flow cytometry. C. An ELISA kit was adopted to measure the secretion of IL-10 in macrophages. D. The mRNA level of CCL24 in macrophages with different treatments was measured by qRT-PCR. E. Western blotting was conducted to detect the protein expression of CCL24 in macrophages (quantified in the right). F. An ELISA kit was used to measure the content of CCL24 in the supernatant of macrophages. Values are the mean ± SD; Student's t-test (Panel: A-C) or Two-way ANOVA (Panel: D-F) with Tukey's post hoc test (Panel: D-F); ** $p < 0.01$.

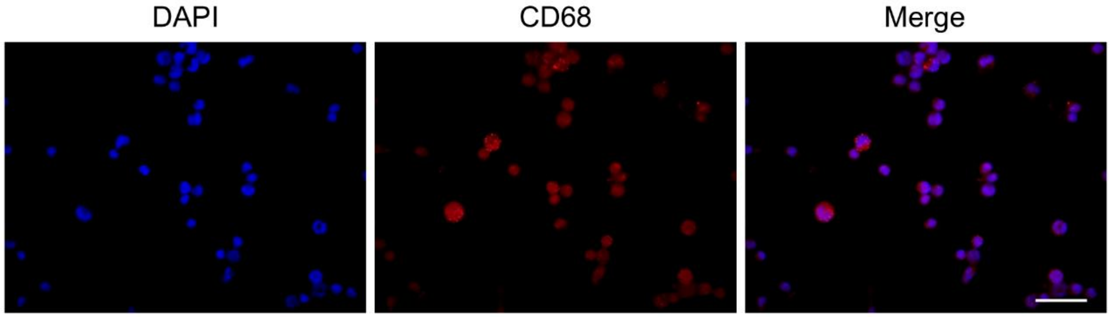

Figure S2. The identification of primary rat peritoneal macrophages

Immunofluorescent detection of CD68 expression in primary rat peritoneal macrophages. Scale bars: 50 μm

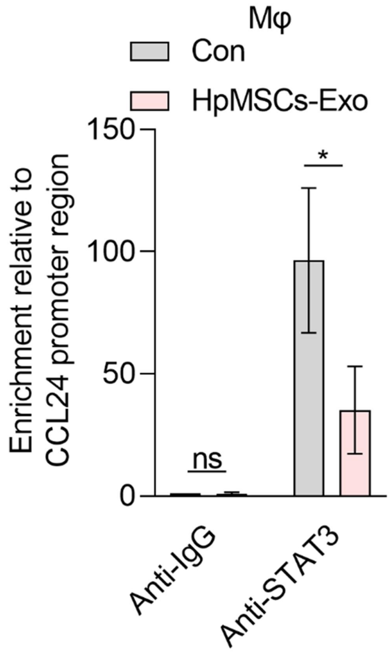

Figure S3. The effects of HpMSCs-Exo on the recruitment of STAT3 to the promoter region of CCL24 in macrophages. THP1-derived macrophages were treated with or without HpMSCs-derived exosomes (HpMSCs-Exo). Then, ChIP-qPCR was adopted to measure the abundance of STAT3 on the promoter region of CCL24 in macrophages. Values are the mean ± SD; One-way ANOVA with Tukey's post hoc test; * $p < 0.05$, ns $p > 0.05$.

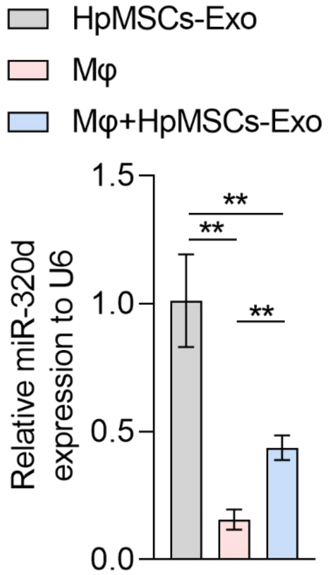

Figure S4. Comparison of miR-320d abundance in native HpMSCs-Exo and macrophages

The miR-320d level in THP1-derived macrophages treated with or without HpMSCs-derived exosomes (HpMSCs-Exo) was measured with qRT-PCR assay. Values are the mean ± SD; One-way ANOVA with Tukey's post hoc test; ** $p < 0.01$.
