# Supplementary figures and images for: Peritoneal MSCs-derived exosomes suppress CCL24 synthesis through miR-320d delivery contributing to the improvement of peritoneal dialysis-associated fibrosis
Source: Sci Rep. 2026 Mar 4;16:11998. doi: 10.1038/s41598-026-42489-w (PMC13069033; doi:10.1038/s41598-026-42489-w)

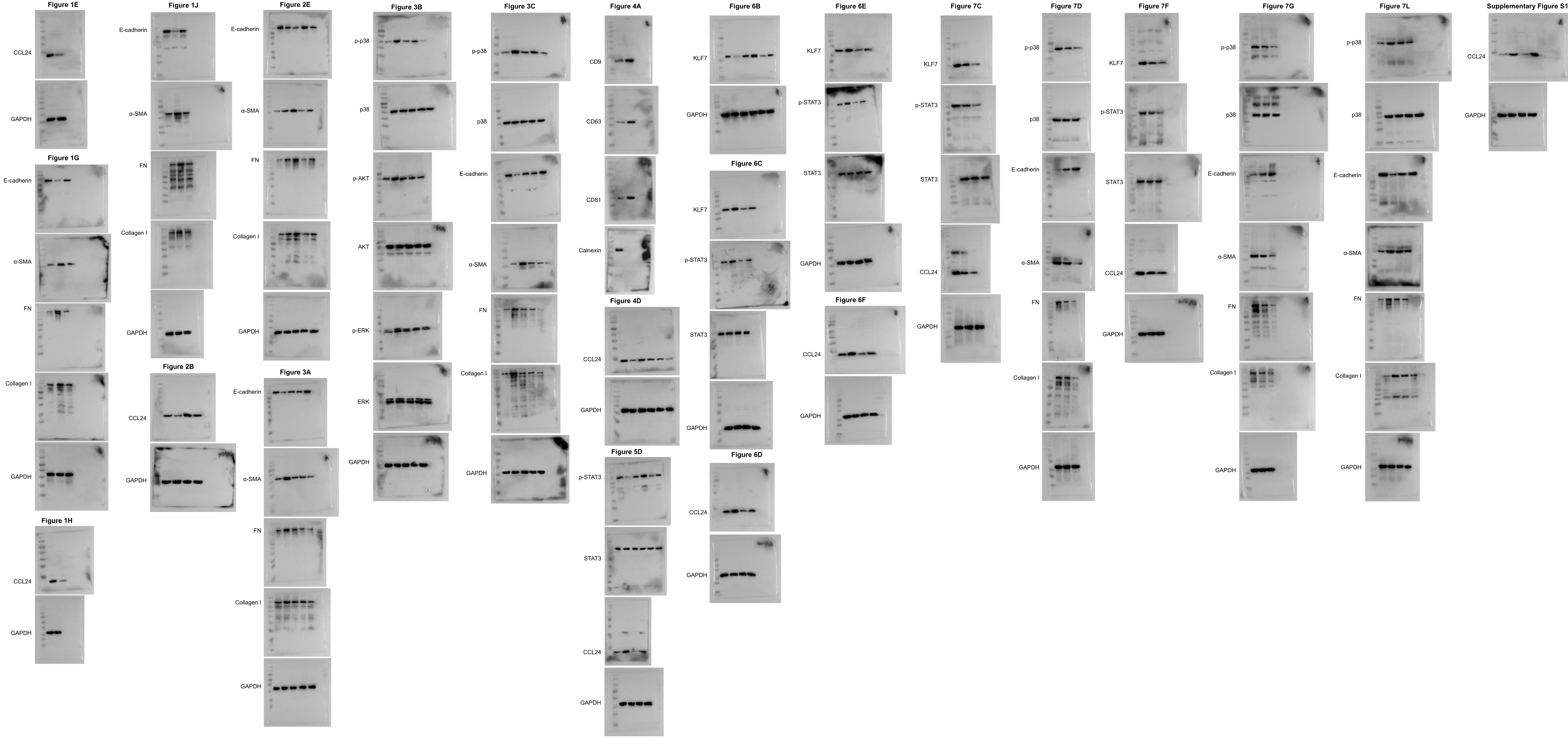

Supplement: Supplementary file 2 — Supplementary Material 2 [file 41598_2026_42489_MOESM2_ESM.pdf]
